# Evaluation of three different laboratory methods to detect preformed human leukocyte antigen antibodies in a South African kidney transplant population

**DOI:** 10.4314/ahs.v21i2.32

**Published:** 2021-06

**Authors:** Luyanda Kwofie, Ronald Anderson, Helen Steel, Pieter Meyer WA

**Affiliations:** 1 National Health Laboratory Service, Immunology Department, Pretoria, South Africa; 2 University of Pretoria, Faculty of Health Sciences, Department of Immunology, Pretoria, South Africa

**Keywords:** Preformed human leukocyte, antigen antibodies, kidney transplant, population, South Africa

## Abstract

**Background:**

Anti-human leukocyte antigen antibodies (anti-HLA) play a crucial role in graft. Detection of anti-HLA, both pre- and post-transplant is a crucial investigation in clinical organ transplantation.

**Objectives:**

Three methodologies for the detection of lymphocytotoxic antibodies were compared to establish which of these is best suited to optimise pre-transplant donor-recipient matching.

**Methods:**

Serum samples from 15 renal transplant patients were tested for the presence of anti-HLA by i) cytotoxic-dependent cross-match (CDCXM), ii) flow cytometric cross-match (FCXM) and iii) Luminex-based donor specific antibody cross-match (DSAXM) method, Confirmatory tests for the presence of preformed HLA antibodies were tested using Luminex methodology.

**Results:**

Two (13%) of the 15 patients had positive HLA Class I antibodies (Ab) using all 3 methods. An additional 2 HLA Class I Ab were identified with FCXM/CDCXM. DSAXM identified 1 HLA Class I positive, not indicated by CDCXM/FCXM.

High HLA Class II positivity (40%), identified by CDCXM, while DSAXM and FCXM identified two and one patients, respectively. CDCXM produced 4 false-positive results confirmed by lymphocyte single antigen (LSA) assay.

**Conclusions:**

The DSAXM method appears to add value in pre-transplantation screening to identify pre-sensitised patients that may not reject the donor graft due to the absence of donor-specific antibodies.

## Introduction

Chronic kidney disease (CKD) is a worldwide public health problem. In this context, renal replacement therapy can be done either by dialysis or organ transplantation. However, dialysis is life-long and is associated with reduced quality of life and increased risk of mortality. Kidney transplantation, on the other hand, offers better survival and quality of life benefit for patients with endstage kidney disease relative to dialysis^1^–^4^.

According to the South African Renal Registry Annual Report of 2015, the total number of patients on renal replacement therapies (RRT) was 10 360. Gauteng province had the highest number of patients at 3238 (958 from public sector and 2280 from the private sector). Of the 10 360 patients, 1 440 (13.9%) were on peritoneal dialysis and 7 529 (72.2%) were on haemodialysis. Of these patients, only 1 391 (13.4%) underwent renal transplantation^5^, underscoring the fact that South Africa has one of the lowest deceased organ donation rates in the world, which is estimated at less than around two-million donations per million population per annum compared to 13 million in UK and over 30 million in Spain^5^–^8^. Comparing RRT to kidney transplantation, the latter results in an improved quality of life, improved social rehabilitation and savings in overall health care costs. Unfortunately, however, the potential benefit of kidney transplantation has not realised its full potential, resulting in awaiting transplant patients remaining on transplant receiving lists for extended periods (between 2–7 years)^9^–^13^.

It is imperative therefore, that available organs are optimally utilised by ensuring that best-practice methods are applied when screening for potential rejection risk. In this context, the impact of detection of pre-formed human leukocyte antigen (HLA) antibodies reactive with transplanted organs is a well-established practice in clinical renal transplantation^14^, ^15^.

Presently, complement dependent cytotoxicity cross-match (CDCXM) remains the most frequently utilised pre-transplant cross-match technique in the South African setting^14^,^15^. However, this technique has limitations due mainly to low viability of cells in both cadaver deceased and living-related donor screening. Although there is still uncertainty in regarding the most sensitive method among the available assays in the routine environment and if they can be used individually. Baranwal and colleagues indicated that Luminex based cross-match predated CDCXM and flow cytometry cross-match results to a reasonable degree, hence, it can be considered the most sensitive in their settings^3^. Therefore, to establish an alternative method for detection of recipient serum antibodies directed against donor antigens is necessary in our country^3^, ^16^–^18^ and represents the primary focus of the current study.

## Methods

### Study Population

Fifteen patients and their living – related donors, who were candidates undergoing their 1^st^ for renal transplantation at the Steve Biko Academic State Hospital and Jacaranda Private Hospital, Pretoria, South Africa, were enrolled in this study, which was conducted from September 2014 through April 2015.

Unfortunately, there is no known details collected at the start of the study regarding potential multiparous or previous transfusions. Written and signed informed consent was obtained from each patient and healthy donor prior to enrolment into the study. The study was approved by the Research Ethics Committee of the Faculty of Health Sciences of the University of Pretoria, South Africa and conformed to good laboratory practice, as well as with the 1964 Helsinki declaration and its later amendments or comparable ethical standards. Ethics certificate reference number: 242/2013.

The patients were cross-matched with their potential donors using three different methods viz the Terasaki microlymphocytotoxicity technique (CDCXM), the flow cytometry cross-match (FCXM) and the Luminex-based donor specific antibodies cross-match (DSAXM).

**CDCXM:** This assay was performed using the standard two-stage National Institutes of Health (NIH) technique (Patel, Terasaki 1969, Peña, Fitzpatrick & Saidman 2013). Donor T- and B-lymphocytes were isolated via attachment to CD2- and CD19-monoclonal antibody-bound immunomagnetic beads respectively, followed by elution (One Lamda, Inc. Hannover, Germany).

Briefly, donor T- and B- lymphocytes were incubated with recipient sera for 30 min at room temperature. Rabbit complement was added to each well and the microplates incubated for 60 min at room temperature. The cells were then stained with a fluorescent dye mix, containing acridine orange (AO) and ethidium bromide (EB) (One Lamda, Inc.) and observed microscopically for cytotoxicity using a fluorescence microscope. DSA (donor specific antigen) binds to the donor cells, activating the complement cascade via the classical pathway, resulting in lysis of lymphocytes detected by differential fluorescence (red - EB, stained dead cells and green - AO stained live cells). CDCXM results for both T- and B-cells were considered positive when cell death exceeded that of the negative control by 20%.

**FCXM:** This assay was performed using patients' serum samples and donor peripheral blood mononuclear cells. The technique was performed by mixing 100µl of a 1x10^6^ cells/ml donor lymphocytes with 20µl of patient or control sera and incubated for 60 minutes at room temperature, followed by three washes with phosphate-buffered saline (PBS; 5min at 600g). To identify alloantibodies and differentiate between T and B cells, the re-suspended pellet was then incubated for 30 min in the dark with 5µl of phycoerithrin-CY5 labelled anti-CD3 (CD3-PC5) (Beckman Coulter, USA), 5µl of phycoerithrin labelled anti-CD19 (CD19-PE) monoclonal antibody (Beckman Coulter, USA), and 10µl of goat F(ab)2 antihuman IgG - fluorescein isothiocynate (FITC) antibody (Beckman Coulter, USA). Two wash steps were then performed as described above and cells analysed following re-suspension in 200µl of PBS.

Multicolour flow cytometric analysis was performed using the Cytomics FC 500 instrument (Beckman Coulter, USA). Viable lymphocytes were gated on the basis of their forward and side- scatter characteristics. Flow cytometry crossmatch results were expressed as the median channel florescence shift from the negative serum of either the T- and/or the B-cells.

**DSAXM:** This assay was performed as per the manufacturer's instructions (Gen-Probe, LIFECODES, Stamford, CT). It is used to detect IgG antibodies to donor-specific class I and class II HLA. Donor lymphocytes were isolated from peripheral blood, solubilised with non-ionic detergents, followed by centrifugation to remove cell debris and the supernatant (lymphocyte lysate) decanted.

Donor lysates were then incubated with capture beads at room temperature for 30 min in the dark with intermittent mixing to facilitate binding of the solubilized donor HLA molecules to the immunofluorescent beads. The assay included control beads to monitor the amount of background fluorescence in the assay.

After incubation, beads/lysate mixtures were transferred to the filter plate and washed. Diluted recipient serum was then added to the beads and incubated with agitation in the dark for 30 min followed by 3 washes. Diluted anti-human IgG streptavidin-phycoerythrin conjugate (SAPE) was then added and incubated for a further 30 min period. After addition of wash buffer, readings were taken on the Bio-Plex array system, Luminex 100 analyser.

Determination if a captured bead was positive or negative for DSAXM was performed according to the manufacturer's instructions. The mean fluorescence intensity (MFI) value of each bead was compared to three cut-off values (background adjustment factor; BAFs). The cut-off values were calculated from the measured background of three negative control beads in each test well. The process was repeated for each of the two beads to obtain three results (Adjusted MFI values). A sample was considered positive if two or more Adjusted MFI Values were positive and a sample was considered to be negative if two or more Adjusted MFI Values were negative^19^.

HLA antibody analysis: In this assay, serum samples from the recipients were analysed for HLA class I and class II IgG HLA antibodies using the commercially available LABScreen mix, single antigen (LSA) class I and class II assay kit (One Lambda, Inc., Canoga Park, CA, USA) on a Luminex platform (Bio-Plex 200, Bio-Rad Laboratories, USA). The procedure was performed according to the manufacturer's instructions. Briefly, serum was first incubated with LABScreen® beads coated with HLA antigens (One Lambda, Canoga Park), using a 96 V-shaped well plate, followed by washing to remove unbound antibodies. Alloantibodies present in the test serum bind to the antigen-coated beads and are detected by addition of R-Phycoerythrin (PE)-conjugated goat anti-human IgG in the second incubation step. All incubations were performed on a gently rotating platform in the dark at room temperature. After the last wash, buffer was added to each well and samples analysed. Trimmed Mean Fluorescence values were recorded using Luminex 100 IS v 2.3 software (Luminex Corporation, USA) for data analysis. Negative and positive controls were included with each assay. Data was normalised to the background value of the negative control serum and a minimum of 50 beads was required for the assay to pass. Data analysis was performed using HLA Fusion 3.0 software (One Lambda, Inc.). To determine the presence of HLA antibodies in the patients' sera, Mean Fluorescence Intensity (MFI) of the bead was normalized against the MFI of the negative control serum. A bead was regarded as being positive if the MFI value was 1000 or higher. This procedure enables detection of the entire spectrum of HLA allo-antibodies in recipient serum.

## Results

The recipient group consisted of 9 (60%) females and 6 (40%) males, while the living related donor group consisted of 6 (40%) females and 9 (60%) males.

The mean age of the recipients was 39.3 (range 21–61 years) with Caucasians accounting for 73%. Of these, the mean donor age was 41.7 (range 23–58 years) with Africans accounting for 27% of this group. Recipients and their potential donors were all blood group compatible. Donor and recipient profiles are shown in Table 1.

**Table 1:** Recipient-donor profile for 15 allograft recipients awaiting kidney transplants in South Africa (September 2014–April 2015)

Variables	Donors (n=15)	Recipients (n=15)
**Age in Years - Mean (SD)**	47.7 (11)	39.3 (13.4)
Gender -n (%)		
Male	9 (60)	6 (40)
Female	6 (40)	9 (60)
**Ethnicity-n (%)**		
Caucasian	11 (73)	11 (73)
African	4 (27)	4 (27)
**Blood Type-n (%)**		
A	5 (33)	7 (47)
B	1 (7)	4 (27)
O	9 (60)	3 (20)
AB	0 (0)	1 (6)
**Medical history (recipients) - n (%)**		
Diabetic mellitus		1(7)
Hypertension		12(80)
Hyperuricaemia		2(13)
*Systemic lupus erythematosus* (SLE)		2(13)
Hypothyroidism		2(13)
Hypercholesterolaemia		5(33)
Unknown		1(7)
Dialysis (HD/PD)		9/6
Average duration (mon)		23.5(10.5)

As shown in Table 2, seven (47%) of the fifteen patients were CDCXM positive (in terms of class I and /or class II), two (13%) patients were class I positive, thirteen (87%) were class I negative, while six (40%) patients were class II positive and nine (60%) were class II negative. One (7%) patient was class I and class II positive.

**Table 2:** Results comparing the three cross-match methods for patients awaiting kidney transplants (September 2014 – April 2015)

	NIH		Luminex		Flow	
	CDCXM		DSAXM		FCXM	
Patient	Class I	Class II	Class I	Class II	Class I	Class II
**1**	N	P	N	N	N	N
**2**	N	N	N	N	N	N
**3**	N	N	N	N	N	N
**4**	N	P	P	P	N	N
**5**	N	N	N	N	N	N
**6**	N	N	N	N	N	N
**7**	P	N	P	P	P	N
**8**	N	N	N	N	N	N
**9**	N	N	N	N	N	N
**10**	P	P	N	N	P	P
**11**	N	P	N	N	N	N
**12**	N	P	N	N	N	N
**13**	N	P	N	N	N	N
**14**	N	N	N	N	N	N
**15**	N	N	N	N	N	N
Total Positive - n (%)	2 (13)	6 (40)	2 (13)	2 (13)	2 (13)	1 (7)
Total Negative - n (%)	13 (87)	9 (60)	13 (87)	13 (87)	13 (87)	14 (93)

N = Negative; P= Positive

**Figure 1:**
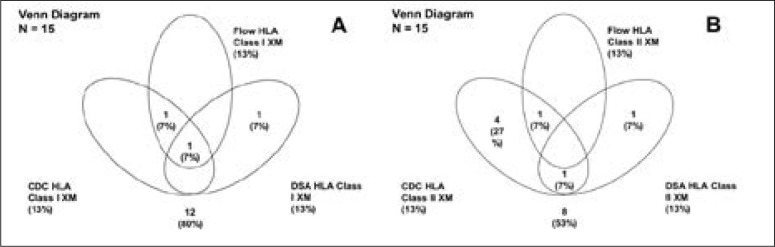
Comparison between cross-match testing methods. CDC – complement-dependant-cytotoxicity; DSA – donor specific antibodies; FCXM – flow cytometric cross-matching

CDCXM, FCXM and DSAXM results performed on all recipients included in this study are presented collectively in Figure 1. For all three methods, two (13%) patients were truly positive for HLA class I (Figure 1A). CDCXM and FCXM results correlated for these patients, but further analysis revealed that DSAXM correlated with only one patient. CDCXM exhibited a high percentage of positive HLA class II (40%), while DSAXM identified only two (13%) patients and FCXM only one (7%). HLA antibody screening using HLA class I and class II single antigens was performed to detect the possible presence of antibodies against the donor antigens (Table 3).

**Table 3:** Lymphocyte single antigen (LSA) detection, panel reactive antibodies and alleles detected on the 4 patients who tested positive by CDC crossmatching

	HLA Class I		HLA Class II	
Patient No:	PRA%	Alleles identified	PRA%	Alleles identified
1	5	A66, B45, B60, B76	2	DPQ1, DPQ5
11	14	A2, B18, B41, B45, B48, B50, B60, B61, B71, B72, B75, B76	0	N/A
12	1	B73	0	N/A
13	0	N/A	0	N/A

%PRA – Percentage of panel reactive antibodies

## Discussion

Renal transplantation is the option of choice in RRT for patients with end-stage kidney disease^20^, ^21^. In South Africa, the CDC pre-transplant crossmatching method is still widely used as a technique of choice in the pre-renal transplantation work-up^14^, ^15^, and is routinely used in our setting for both deceased and live donor pre-transplantation. It is an important tool in evaluating histocompatibility in recipient and donor matching. A negative CDCXM pretransplantation is known to reduce the risk of hyperacute rejection significantly^22^, with the presence of HLA antibodies being indicated by a positive CDCXM.

The presence of these antibodies, especially DSA, plays a crucial role in graft rejection and eventually graft loss^23^–^26^. However, the CDCXM is known for its lack of sensitivity, and limitations, including both false negative and -positive results. False-negative results may occur if there are low levels of DSA or if the antibody isotype does not activate complement. In this context, HLA antibodies of the IgG2 and IgG4 subclasses are unable to activate complement cascade and consequently will not be detected^21^, ^27^, ^28^. In addition, CDCXM is also known to be reactive with non-specific antibodies (i.e. IgM antibodies) leading to false-positive reactions, hence more specific and sensitive techniques are required^2^, ^21^, ^29^. Our results showed a 40% false-positivity rate for HLA Class II, which confirms the lack of sensitivity of this procedure. The results suggest that the CDCXM HLA class II method has limited diagnostic application because of this high false-positivity rate. On the other hand, CDCXM HLA class I testing, which has limited application in cadaver deceased donor transplant, may be beneficial in living related donor transplant when used in conjunction with another more sensitive cross-match method.

Luminex DSAXM is a novel procedure in our local setting. To our knowledge, this procedure is not widely used for routine or diagnostic purposes in tissue immunology centres in South Africa, making our study novel in this regard.

Although DSAXM Class II false results have occasionally been reported^30^–^33^, in our study, the DSAXM results for HLA class II correlated well with FCXM results. Although our sample size was small, DSAXM was preferred to the CDCXM procedure, thereby eliminating the reliance on viable cells because DSAXM uses donor lysate, which can also be stored for future analysis. In addition, DSAXM detects only IgG DSA, and is therefore more sensitive^17^,^34^, ^35^.

The DSA and FCXM methods showed equivalent performance. However, one patient with a positive FCXM class II reaction was negative with DSAXM. It was established that this patient was pre-sensitised, as the presence of HLA class II antibodies were detected as per LSA as shown in Table 3, but did not have specific antibodies to the donor's HLA Class II antigens. We therefore conclude that despite the small size of our study group, the preliminary results shown in this study demonstrate that, DSAXM could add potential value in pre-sensitised kidney transplant patient screening in order to identify those pre-sensitised patients that may not reject the donor graft due to the absence of donor-specific antibodies. It was also established that CDCXM (HLA Class II) could be excluded in favour of more sensitive methodologies such as, FCXM, DSAXM or LSA.

## Limitation of the study

The main limitation of this study was the small numbers of subjects recruited overall, particularly for the post-transplant study. One of the reasons being that most recipients were not residing in Gauteng in close proximity to the hospitals.

Hence, they were unavailable for follow-up study. Overall, the time period for this study was also limited being a post-graduate study set within a timeframe of 2–3 years and the fact that the Greater Tshwane transplantation institutions do limited LRD transplantations. Nevertheless, potentially interesting findings with translational potential resulted from the study.

There have been reports by others of false-positive DSAXM Class II results^30^–^33^. However, the DSAXM Class II results recorded in the current study correlated well with FCXM, as well as with the Luminex DSA method. This is one of the major issues for future study. In our study, the HLA groups/ isotopes of interest were HLA, B, C, DR and DQ. However few case reports have demonstrated anti-HLA DP as having detrimental outcomes for renal graft survival^36^, ^37^. As methods improve to identify HLA antigen specificities, the allocation of organs to recipients will become more onerous. Pre-transplant immunological risks remain a major stumbling block and more research still needs to be conducted to improve donor-recipient matching.
